# Percutaneous treatment of IVC obstruction due to post-resection hepatic torsion associated with IVC thrombosis

**DOI:** 10.1186/s42155-019-0056-2

**Published:** 2019-04-25

**Authors:** Thuong G. Van Ha, Thomas G. Tullius, Rakesh Navuluri, J. Michael Millis, Jeffrey A. Leef

**Affiliations:** 1 Department of Radiology, University of Chicago Medical Center, 5841 South Maryland Avenue, MC 2026, Chicago, IL 60637 USA; 2 Department of Surgery, University of Chicago Medical Center, 5758 South Maryland Avenue, Chicago, IL 60637 USA

**Keywords:** Hepatic venous outflow obstruction, Living donor liver transplantation, Thrombectomy, Venous stenting

## Abstract

**Background:**

Migration of the left hepatic lobe into the potential space following right lobe resection can result in torsion and hepatic venous outflow obstruction with compromised venous return from the IVC. If untreated, significant morbidity and mortality can develop.

**Case presentation:**

We report a case of a 29-year-old female with Lynch syndrome who underwent right lobe resection for a metastatic hepatic tumor. There was subsequent migration of the liver remnant, torsion of the IVC, and impaired hepatic outflow, successfully treated with thrombectomy and stenting.

**Conclusion:**

Following right hepatectomy, hepatic venous outflow obstruction should be consdered in the setting of hepatorenal failure and hemodynamic instability. Endovascular stenting is a viable treatment option.

## Background

Post-operative venous complications after hepatectomy have been described in the literature and seem to be uncommon (Sato et al., 2012; Mulé et al., 2015). While hepatic torsion causing hepatic venous outflow obstruction (HVOO) is a known complication in living donor liver transplantation (LDLT), only a few reported cases exist in the literature related to post hepatic resection (Umehara et al., 2012; Sato et al., 2014). Relative preservation of anatomic support in hepatic resection compared to LDLT may contribute to this difference. We report a case of successful percutaneous treatment of IVC torsion after right hepatic lobe resection.

## Case presentation

**Fig. 1:**
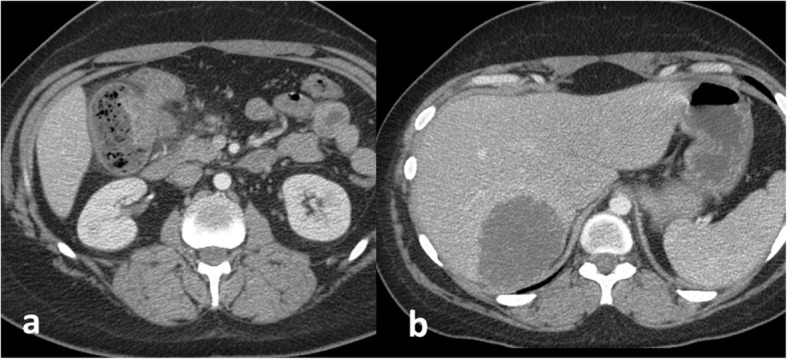
29-year-old female with Lynch Syndrome presents with abdominal pain and bloody diarrhea. **a**. Axial contrast enhanced CT image of the abdomen demonstrates a partial bowel obstruction secondary to a primary colonic neoplasm at the hepatic flexure with surrounding inflammatory changes suggestive of colitis. **b**. Large right hepatic mass measuring up to 8 cm consistent with metastatic disease

**Fig. 2:**
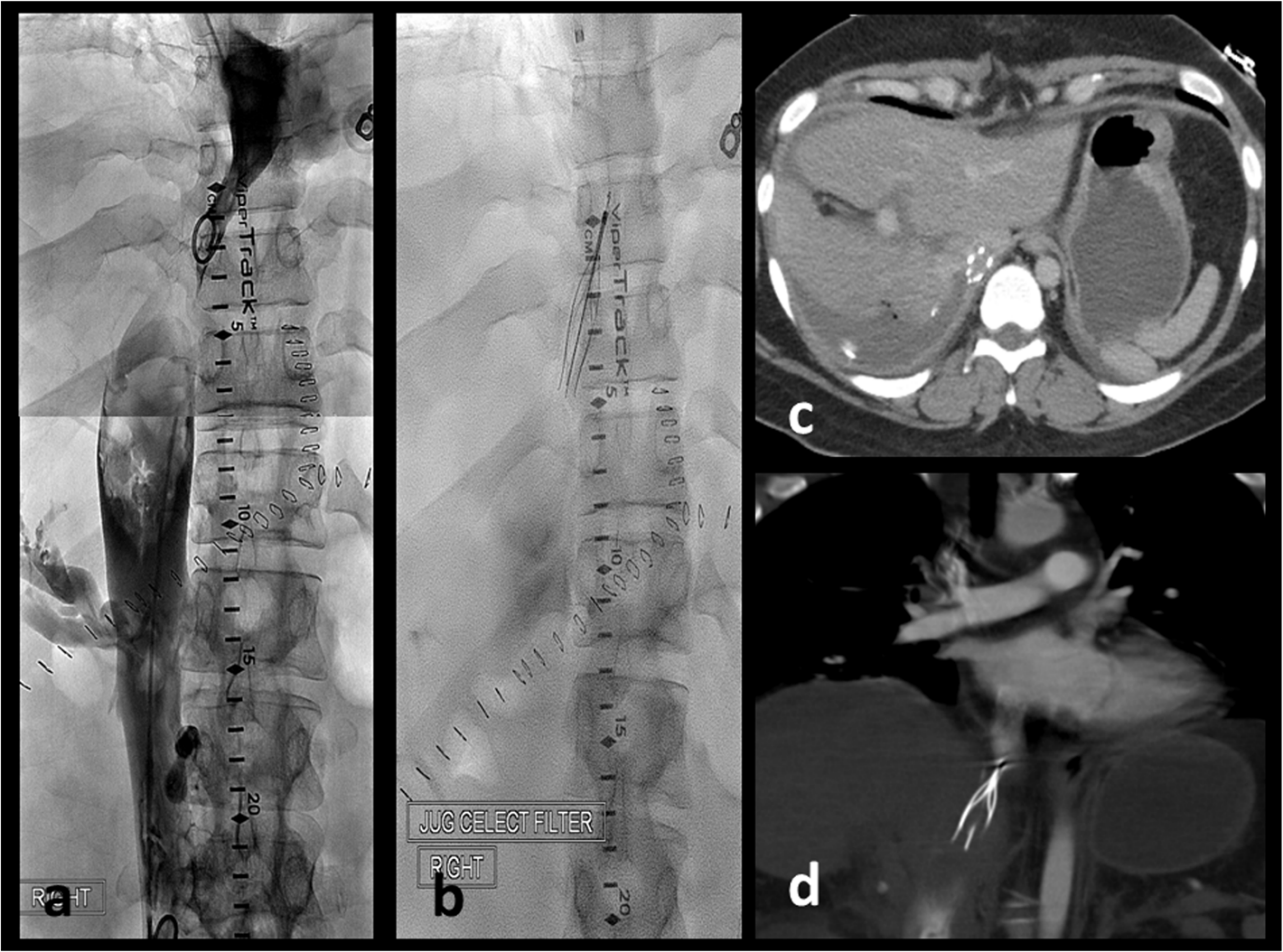
Two days following right hepatic resection, the patient developed deep vein thrombosis. **a**. Venogram demonstrates large clots in the suprarenal IVC and narrowing of the superior aspect of the IVC above the hepatic vein inflow. **b** Suprarenal IVC filter was placed superior to the thrombus. **c**. and **d**. Subsequent contrast enhanced axial CT images of the abdomen shows the liver remnant shifted towards the right into the space vacated by the right hepatic lobe. IVC was severely narrowed at the point above the hepatic vein inflow associated with thrombus in the suprarenal IVC

**Fig. 3:**
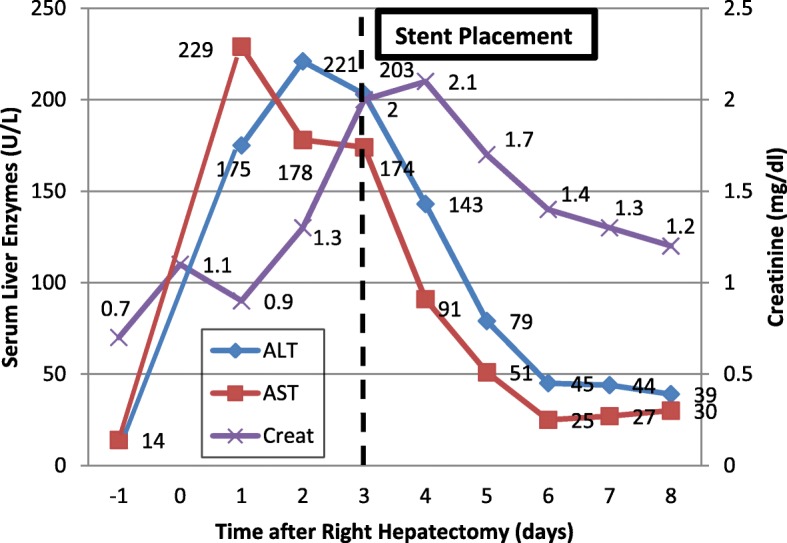
Peri-operative laboratory data. Gradual elevation of the liver enzymes and creatinine levels were observed following right hepatectomy with resolution folllowing IVC stent placement

**Fig. 4:**
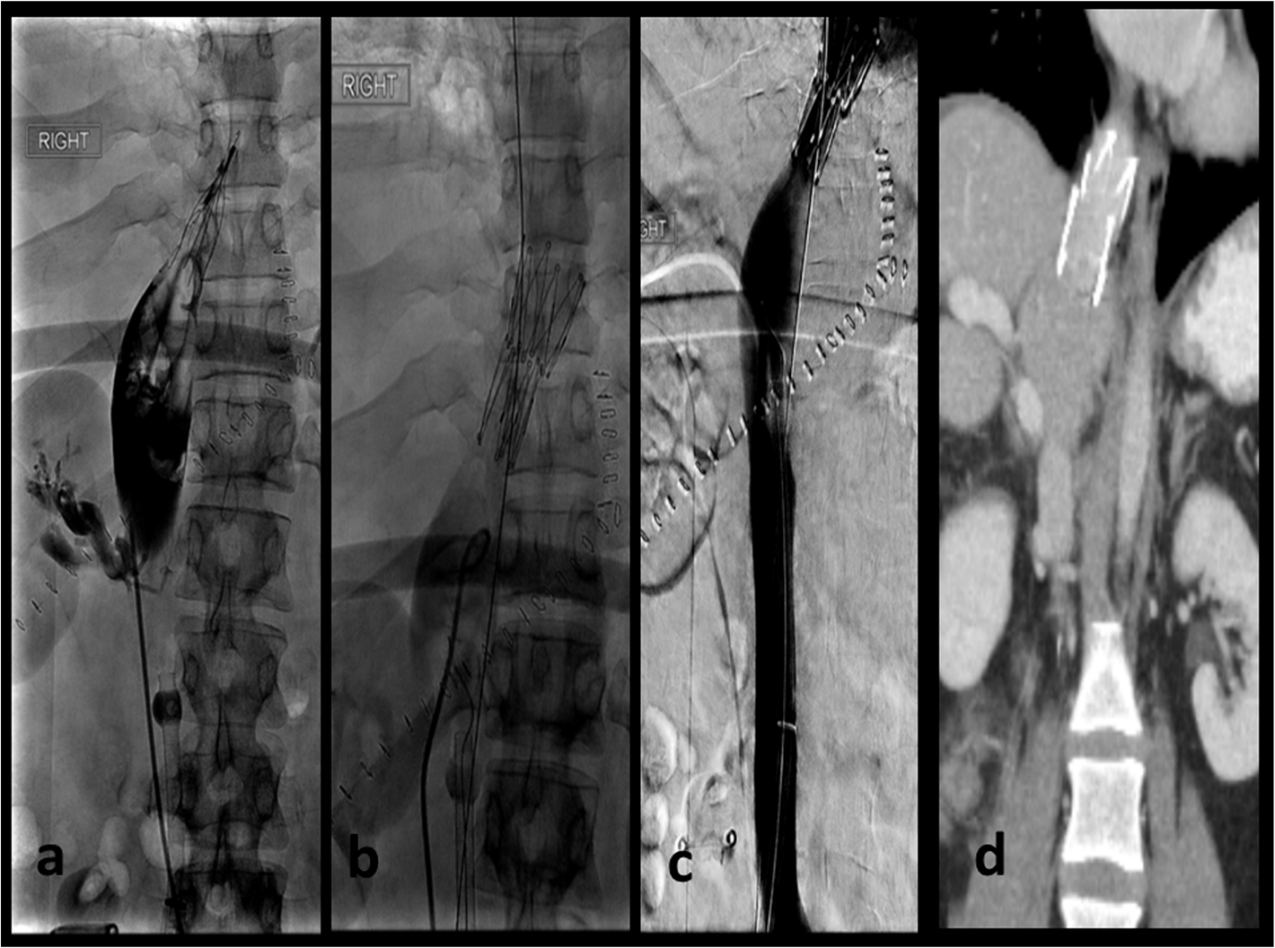
After filter placement, the patient was hypotensive with oliguria and elevated liver enzymes. CT showed extensive thrombus in the IVC. **a**. and **b**. Venogram demonstrated large clot burden in the IVC distal to the filter with AngioVac thrombectomy device in place. Z stent was placed at the site of the kinked area. **c**. and **d**. Venogram following Z stent placement showed a widely patent IVC. Coronal contrast enhanced CT image of the abdomen demonstrates a patent stent in the intrahepatic IVC

A 29-year-old woman presented with abdominal pain, vomiting, and bloody diarrhea. Contrast-enhanced CT of the abdomen showed a partial bowel obstruction secondary to a large proximal colonic mass thought to be a primary tumor [Fig. 1]. In addition, there was a large right hepatic mass suspicious for metastasis. Colonoscopy demonstrated a partially obstructing lesion at the hepatic flexure and biopsy showed moderately-to-poorly differentiated infiltrating adenocarcinoma without mucinous features. Further genetic testing showed loss of MLH1 and PMS2 with mutations in MLH1 and CDH1, suggestive of Lynch syndrome. The liver mass enlarged despite 5 cycles of chemotherapy with FOLFIRINOX. Three months after presentation, the patient underwent a right hepatic lobectomy (with preservation of the left triangular ligament), a cholecystectomy, and diverting ileostomy in preparation for a right hemicolectomy which occurred three months later. Two days later, she presented with deep vein thrombosis. She was placed on intravenous heparin and an IVC (inferior vena cava) filter was requested. During filter placement, there were large clots in the suprarenal IVC and narrowing of the superior aspect of the IVC above the hepatic vein inflow [Fig. 2]. The filter was placed superior to the thrombus. Next day, the patient was found to be hypotensive with blood pressure of 80–90/50–60 mmHg, heart rate of 70–80 bpm, oliguria, and elevated liver enzymes [Fig. 3]. Her hemoglobin remained stable. Given persistent hypotension requiring vasopressors, CT of the abdomen was obtained to evaluate for hemorrhage. The liver remnant had shifted into the space vacated by the right hepatic lobe and the IVC was severely narrowed at the point above the hepatic vein inflow associated with thrombus in the suprarenal IVC. There was concern that venous return was impaired by both the IVC narrowing and IVC clots, and thrombectomy was performed. Initial procedure included IVC thrombectomy using AngioVac (AngioDynamics, Inc., Queensbury, NY) with technical success. However, rethrombosis of the IVC occurred. Review of the imaging showed torsion of the IVC at the level of the subdiaphramatic vena cava near the confluence of the hepatic veins and the IVC causing thrombosis. The patient was retreated with thrombectomy with AngioVac followed by removal of the suprarenal IVC filter and subsequent stenting of the kinked area using a Z-stent measuring 25 × 50 mm (Cook, Inc., Bloomington, IN) [Fig. 4]. Post-procedure, the patient’s blood pressure, liver enzymes, bilirubin, and creatinine returned to baseline levels. The patient was discharged 6 days later on oral rivaroxaban.

## Discussion/conclusions

HVOO is rare after hepatectomy and more common after LDLT, with incidence at 0.1% and 5–13%, respectively (Umehara et al., 2012; Shimahara & Yamaoka, 2002). In LDLT, the occlusion usually occurs at the hepatic vein anastomotic site. In the nine reported cases of post-hepatic resection HVOO, there were five right hepatectomies, three right hepatic trisegmentectomy, and one posterior segmentectomy (Sato et al., 2014; Sequeira et al., 1981; Pitre et al., 1992; Paineau et al., 1993; Poon et al., 1998; Benesch et al., 2002; Wang et al., 2010; Di Domenico et al., 2013). Tumor size is considered a risk factor for post-resection HVOO, ranging from 18 to 21 cm. Due to the large tumor size, the vacated spaces were large enough for the remnant liver to migrate and cause torsion of the hepatic venous pedicle and/or IVC. In these cases, treatment consisted of surgical repositioning and fixation in six patients and endovascular stent placement in two patients. One patient had recurrent torsion following surgical fixation and was treated successfully with stenting. In the three cases involving stenting, patients presented with a slowly progressive clinical course, stent placement was successful, and all survived. In two cases, the kinked left hepatic vein was stented and in the other case the compromised IVC was stented. However, in these cases, extensive IVC thrombus was not present as in our patient. Primarily addressing the thrombus is important as it can result in pulmonary emboli, IVC thrombosis, and clot propagation. Given the recent surgery, thrombolysis was contraindicated. Mechnical thrombetomy of the central veins is technically limited by the small caliber of many commercailly available devices. We selected the AngioVac thrombectomy device given the large diameter of the aspiration catheter and the utilization of a venovenous bypass circuit to treat the significant caval clot burden without substantial hemorrhage (Resnick et al., 2016). The underlying IVC narrowing had to be managed by stenting with a large caliber stent with adequate radial force to overcome the torsion caused by the migrated liver remnant. Only after IVC stenting to address the IVC torsion did our patient stabilize. Long-term patency of stenting in the setting of post-resection HVOO is unknown. In our patient, followup imaging demonstrated patency at 24 months on rivaroxaban alone. No secondary interventions were necessary to maintain stent patency.

Some authors believe preservation of the left triangular ligament is inadequate to prevent left liver lobe dislocation after right hepatectomy (Sato et al., 2014). Others suggest surgical fixation of the falciform ligament to the anterior abdominal wall be performed to prevent left lobe migration (Ogata et al., 2005). Early recognition and intervention is key to preventing mortality. Of the nine reported cases, two fatalities resulted from hepatorenal failure from HVOO. Fatalities were attributed to delayed diagnosis and intervention, with surgical intervention performed at seven days and 10 weeks after hepatectomy, respectively.

Elevated liver enzymes are to be expected in patients with HVOO. Sato, et al. reported significant increase in ALT, AST, bilirubin. Serum creatinine was also elevated. These values normalized after corrective surgery (Sato et al., 2014). In our patient, ALT and AST were elevated more signifincantly than the bilirubin level. Serum creatinine was also increased. These normalized after endovascular treatment. Despite its relatively uncommon incidence, HVOO should be consdered in the setting of hepatorenal failure and hemodynamic instability after right hepatectomy and can be successfully treated endovasculalry.

## Abbreviations

HVOO: Hepatic venous outflow obstruction
IVC: Inferior vena cava
LDLT: Living donor liver transplantation
